# Safety of up to 140 Daily Applications of Recombinant Human Platelet‐Derived Growth Factor (rhPDGF‐BB) Onto Skin Wounds: Unboxing the Evidence

**DOI:** 10.1111/wrr.70108

**Published:** 2025-11-11

**Authors:** Herbert B. Slade, Samuel E. Lynch, Jaime E. Dickerson

**Affiliations:** ^1^ Chisholm Clinical Research Services Fort Worth Texas USA; ^2^ Lynch Regenerative Medicine Franklin Tennessee USA; ^3^ Coral Snake Consulting Fort Worth Texas USA

**Keywords:** becaplermin, boxed warning, Regranex, rhPDGF‐BB, skin, wound healing

## Abstract

Recombinant human platelet‐derived growth factor BB (rhPDGF‐BB), is the only growth factor approved by the US Food and Drug Administration (FDA) for tissue regeneration and rejuvenation indications. It has received four FDA approvals for both soft tissue (e.g., skin) and hard tissue regeneration/rejuvenation. Regranex gel, 0.01% rhPDGF‐BB, is the only growth factor approved by the FDA for the promotion of skin wound healing. While the safety of one and two 15 g tubes of Regranex, generally sufficient for up to 60 daily applications onto open skin wounds, has never been questioned, a decade after its introduction in 1997, a boxed warning regarding rates of cancer mortality was placed on its label for daily use of three or more tubes. This was based on a mathematical calculation on incomplete data from an insurance claims database which was subsequently invalidated with the addition of three more years of data. Removal of this warning from the label required another decade and several very large propensity‐matched database studies including over 13,000 patients. These studies provided incontrovertible proof that up to 140 daily applications (≥ 4 tubes) of rhPDGF‐BB onto open skin wounds are safe, with no increased risk of either cancer development or cancer mortality. Removing a boxed warning is an arduous task that requires extensive and robust evidence; less than 4% of box warnings are removed once placed. Thus, the successful removal of the boxed warning from the Regranex label should reassure both prescribers and patients of the product's safety.

## 1 Introduction

**FIGURE 1:**
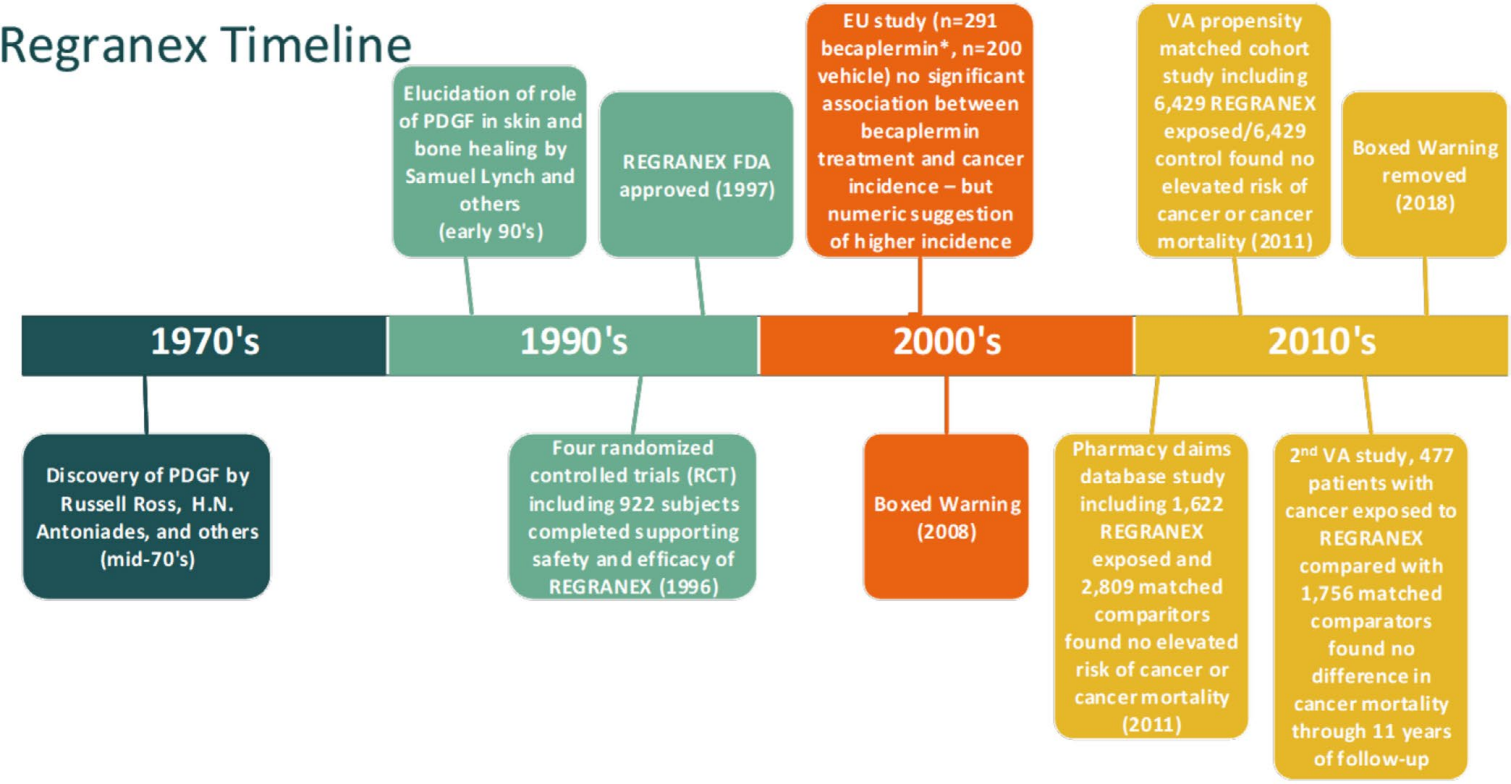
Timeline of major milestones in the development and subsequent marketing of Regranex (rhPDGF‐BB) gel. *A non‐Regranex becaplermin formulation was used in the EU study.

Recombinant human platelet‐derived growth factor BB (rhPDGF‐BB), is the first and only growth factor to be approved by the US Food and Drug Administration (FDA) for tissue regeneration and rejuvenation. Regranex, a 0.01% rhPDGF‐BB gel, is the only growth factor FDA approved to promote the healing of skin wounds, specifically non‐ischemic, neuropathic skin ulcers in the lower extremities of diabetics (commonly referred to as diabetic foot ulcers, or DFUs). In the nearly three decades since its introduction in 1997 rhPDGF‐BB (generic name becaplermin) has been extensively studied; as of this writing there are at least 122 publications in the peer‐reviewed literature accessed through PubMed using ‘becaplermin’ or ‘Regranex’ as Title/Abstract search terms. Its popularity is partly because pure platelet‐derived growth factor (PDGF) is the only tissue growth factor proven in randomised controlled clinical trials to be safe and effective for promotion of tissue regeneration. There have been no other drugs or biologics approved for stimulation of wound healing since the Regranex approval [1]. Moreover, pure PDGF has a well‐established mechanism of action that leads to improved healing, including the enhancement of extracellular matrices such as collagens, hyaluronic acid, elastin and fibronectin and revascularization of the skin and other tissues [2, 3]. While rhPDGF‐BB has been widely studied and utilised clinically to promote skin healing, misinformation persists regarding its safety for long‐term daily application onto skin wounds (Figure 1). This misinformation is often the result of erroneous interpretation of information produced by search engines relating to its link to cancer, with the artificial intelligence tools presenting molecular biology studies that show *natural PDGF production is elevated as a result of cellular transformation, but also showing that addition of exogenous PDGF cannot cause the transformation of cells*. In other words, addition of PDGF has never been demonstrated to cause cancer (i.e., transformation of normal cells to cancer cells), but if a cancer develops as a result of a mutagenic or carcinogenic event certain types of transformed cells may produce elevated levels of PDGF and its receptors as well as elevated levels of many other proteins. This critically important distinction has been further confused by the boxed warning placed on the Regranex label in 2008, despite its removal 7 years ago as of this writing. That warning related specifically to the long‐term daily use of Regranex on chronic full‐thickness skin wounds, and was proven erroneous through 10 years of collaborative research; even the long‐term daily use of Regranex on skin wounds is safe. This review summarises the comprehensive series of investigations and over a decade of clinical evidence that supports the safe long‐term daily use of rhPDGF‐BB to promote the healing of skin wounds.

### 1.1 rhPDGF‐BB

PDGF was one of the first growth factors discovered and is a keystone component of normal wound healing. PDGF is an approximately 25–30 kDa dimer of two disulfide‐linked polypeptide chains; a short chain of approximately 14.5 kDa (A chain) and a longer chain of between 16 and 17.5 kDa (B chain). Both the homodimers (AA, BB) and the heterodimer are physiologically active, with the BB isoform considered the most active for applications in wound healing and regenerative medicine [4, 5]. Becaplermin (rhPDGF‐BB) consists of BB homodimers.

The idea that exogenously applied growth factors could stimulate skin healing and rejuvenation including new extracellular matrix production (e.g., collagens, hyaluronic acid, elastin, etc.) and epithelialization was the logical extension of the natural expression of growth factors such as PDGF‐BB in response to injury, and the stimulatory effects of PDGF‐BB on reparative cell proliferation (mitogenesis), chemotaxis and extracellular matrix production as well as angiogenesis [6]. Moreover, evidence indicates that the level of growth factors is decreased in certain types of wounds, and in certain patients (e.g., older or physiologically compromised). For example, fluid obtained from acute wounds supported mesenchymal cell proliferation and keratinocyte migration, while chronic wound fluid did not [7]. Quantitative analysis of cytokines, growth factors and proteases in wound exudates showed clear differences, the poorly healing chronic wound milieu being highly proteolytic and with generally much lower levels of growth factors than those for healing acute wounds [8, 9, 10].

The availability of recombinant human growth factors permitted studies in which various exogenous growth factors could be added to skin wounds to evaluate their effects on healing [11]. Despite encouraging results in animal models, clinical results with single growth factors have been, for the most part, disappointing with the sole exception of rhPDGF‐BB [12, 13, 14]. Only recombinant human PDGF‐BB has received marketing authorization from the US FDA, first for the promotion of healing of skin wounds in diabetic patients in 1997, followed by approval for the promotion of regeneration of intra‐oral tissues in 2005 and regeneration of bone and surrounding tissues in orthopaedic applications in 2015 and again in 2018 [15, 16]. More recently, the use of pure PDGF has also become popular in certain medical aesthetic procedures and for promoting healing following plastic or dermatologic surgery.

## 2 rhPDGF‐BB (Regranex) gel Clinical Trials for Safety and Efficacy

Six skin healing clinical trials were included in the Biologics License Application (BLA) for Regranex. Two of these were Phase 1 or Phase 2 studies which nevertheless contributed to the overall safety database. Four randomised, controlled, prospective clinical trials were used to establish efficacy and included 922 patients, 478 treated with either the 30 μg/g (0.003%) (*N* = 193) or the 100 μg/g (0.01%) (*N* = 285) doses. Characteristics of the patient populations and of the eligible DFU were similar across all four studies. Patients were adults with type 1 or type 2 diabetes mellitus and at least one but not more than three neuropathic, adequately perfused, full thickness DFU. If more than one ulcer was present, the largest was selected as the study ulcer. Ulcers had to have been present for a minimum of 8 weeks and at least 1 cm^2^ in area. The upper area limit varied between 10 and 100 cm^2^ depending on the study. The results of these studies have been published individually [17, 18, 19], as part of a meta‐analysis [20], and in several reviews [21, 22] and will not be discussed in detail here. The dose ultimately selected for marketing, 0.01%, had a consistently greater incidence of complete wound closure and a consistently shorter time needed to achieve complete healing than either vehicle control or standard care.

The overall safety profile for Regranex that emerged from the registration trials was based primarily on the 922 patients (478 exposed to Regranex) from the randomised, blinded trials in patients with a DFU. No significant safety concerns were uncovered based on an analysis of all reported adverse events. The theoretical possibility of an increase in neoplastic events resulting from treatment with becaplermin given its mitogenic properties was of concern from the outset of these trials and was specifically monitored. There were in fact 11 occurrences of neoplasms. These were of various types and origins and were evenly balanced between the Regranex and comparator treatment groups [23]. None were associated with a lower extremity. In the clinical review of the Regranex BLA the FDA reviewer noted that ‘theoretical concerns raised by the biology of PDGF (i.e., increased vascular events or neoplasms) have not been confirmed by the clinical studies; the drug is in general well tolerated; product discontinuations, infectious adverse events, tumorigenicity, cardiovascular problems, and deaths were similar between standard care, vehicle and product treatment arms’ [23].

## 3 The Boxed Warning

The known mitogenicity of PDGF, the sequence homology of the PDGF gene and the c‐sis oncogene, and the association of continuous, exaggerated autocrine or paracrine expression of PDGF and its receptors by previously transformed tumour cells had heightened the level of vigilance on the part of the Sponsor and regulators for any indication that topical application would increase the incidence of neoplasms [24]. As noted previously, no such signal was observed during the randomised controlled clinical trials. A 2‐year follow‐up including 75% of patients from two placebo‐controlled European trials of an ineffective, sterilised formulation also found no correlation between daily treatment with this becaplermin formulation and the incidence of cancer. However, European regulators noted that of the 10 new cancers, eight (2.7%) were in the 291 patient becaplermin group and two (1%) in the 200 patient vehicle/standard of care control group (RR = 2.8, 95% CI 0.6, 12.8). None of the reported cancers were in close proximity to the treated ulcer [25]. Critical weaknesses of this follow‐up study were small size, incomplete follow‐up and the relatively short time period. While the potential signal of increased risk of incident cancer was not statistically significant, the European Medicines Agency (EMEA, now EMA) felt that it warranted further exploration. Ziyadeh et al., therefore, carried out a propensity‐matched retrospective cohort study utilising an insurance claims database (United Healthcare), including 1622 diabetic patients who had no known cancer, and had been prescribed Regranex (assumed to be exposed), matched with 2809 comparator patients similar with the exception of Regranex prescriptions, plus 480 exposed patients who could not be matched [26]. The study was planned to follow patients who had initiated Regranex between 1998 and 2003, continuing to follow patients until disenrollment, death or 31 December 2003. With a mean of 20 months of follow‐up at the conclusion of the study, it was reported that there was no increase in the risk of new cancers (RR = 1.2, 95% CI 0.7, 1.9). Cancer deaths had been ascertained by submitting the roster of matched comparators, matched and unmatched becaplermin initiators to the National Death Index, resulting in the identification of 16 cancer deaths (8 among PDGF initiators, 8 among non‐PDGF comparators). Overall, cancer mortality for Regranex initiators was not different from non‐PDGF treated comparators (RR = 1.8, 95% CI 0.7, 4.9). Although the total number of cancer deaths was insufficient to test for a dose response, an ad hoc cut of the data at three or more dispensings of 15 g tubes, generally sufficient for 60–90 daily treatments, suggested a potentially higher rate of cancer deaths for the exposed cohort (RR = 5.2, 95% CI 1.6, 17.6) [25]. This estimate was calculated on 4 cancer deaths in the Regranex group. Despite the wide confidence intervals, the fact that the cohorts had been selected specifically from among patients with no known malignancy, the fact that there was only a single person who died of cancer after being prescribed three tubes of Regranex, and the fact that no risk was associated with four or more tubes, the point estimate was sufficiently large to raise concern with the FDA, resulting in a boxed safety warning which extrapolated the concern to patients with known malignancy [27]. In contrast, the EMEA found the same data to be inconclusive, asking that a new pharmacoepidemiologic study be conducted (VA study #1).

## 4 Removing the Box

The FDA directs companies to add boxed warnings to a drug or biologic label when there is plausible information that links use of the drug or biologic to a significant risk. Over the last decade there were 65 new boxed warnings added to the existing 151, with just nine removed [28]. While there is no established policy or set of rules for the removal of a boxed warning, it is understood that the bar for removing a warning is much higher than for placement [29].

**FIGURE 2:**
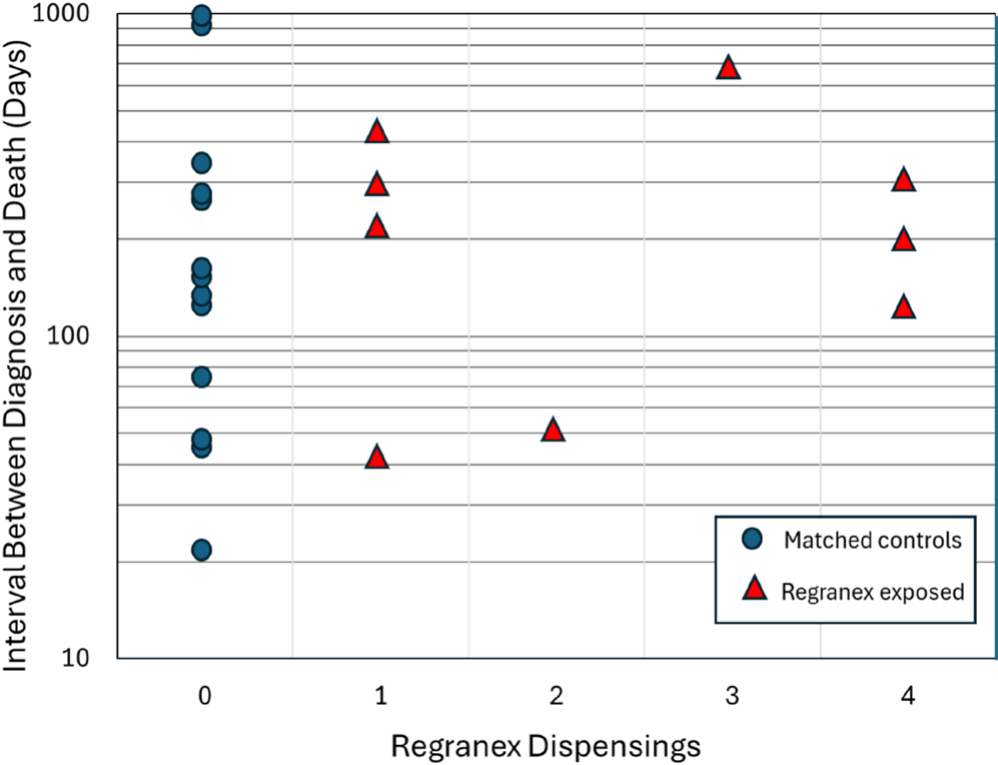
Interval in days between cancer diagnosis and death for propensity matched controls (blue circles), and patients exposed to 1–4 tubes of Regranex (red triangles). Mean and median time to death (days): 245, 142 for control (0 tubes); 257, 212 all Regranex; 324, 247 (3 or 4 tubes). Regranex exposed patients had longer average and median times between cancer diagnosis and death than non‐exposed controls.

In their 2011 publication Ziyadeh et al. repeated their prior analysis using more extensive follow‐up data including up to 6 years for cancer incidence and up to 9 years for cancer mortality. Consistent with the earlier analysis, there was no increased risk of incident cancer (RR = 1.2, 95% CI 0.7, 1.9). Moreover, the rate ratios for cancer mortality were substantially reduced and not statistically significant when considering either all Regranex patients (RR = 1.1, 95% CI 0.5, 2.4) or just those exposed to three or more tubes (RR = 2.4, 95% CI 0.8, 7.4) [26]. Of note, when the interval between cancer diagnosis and death was assessed based on the number of Regranex tubes dispensed (1, 2, 3 or 4), there was no shortening of life or dose response (Figure 2). On the contrary, the median interval between cancer diagnosis and death was actually 75% longer for patients dispensed three or four tubes of Regranex compared to non‐exposed controls. The updated information narrowed the range of plausible point estimates for the association between cumulative use (3 or more dispensings) of Regranex and cancer mortality so that the 95% CI for the revised estimate included the null value—indicating that the potential association could be explained by chance alone [26].

As requested by the EMEA, a more robust study was conducted by the Center for Healthcare Organization and Implementation Research, in association with the US Veterans Affairs (VA) hospital in Bedford, MA. This very large retrospective propensity‐matched cohort study utilised the VA electronic medical record database of approximately 10,000,000 patients, more than 2 million of whom were diagnosed as diabetic. The rhPDGF‐BB cohort included 6429 patients with no known cancer and a DFU, who had been in the VA system for at least a year and who had been dispensed Regranex between 1999 and 2007 [21, 26]. The propensity‐matched cohort (1:1, 6429) was selected from among 893,344 diabetic patients who had never been exposed to becaplermin. It should be noted that becaplermin usage was relatively high for the exposed cohort; 37% were dispensed at least 3 tubes, and 16%, 5 or more tubes, sufficient for approximately 100–150 days of daily dosing [30]. The study found no evidence for an increase in risk of cancer incidence or cancer death with Regranex use.

There were 197 cancer deaths in the Regranex cohort (3.1%), and 206 cancer deaths in the matched comparator cohort (3.2%) with the hazard ratio 0.9 (95% CI 0.8, 1.2) [25]. Analysis by sub‐group (including prior amputation, peripheral neuropathy, insulin use, and various demographics) or by cancer type also failed to show any elevated risk. For the subgroup dispensed three or more tubes, the hazard ratio for cancer mortality was 1.0 (95% CI 0.7, 1.5) [25]. In a smaller cohort (1507 becaplermin exposed, 1507 unexposed), the hazard ratio for incident cancers was 1.1 (95% CI 0.8, 1.4) [25]. These risk estimates were all close to 1.0 with narrow confidence intervals, providing a more stable estimate than the Ziyadeh et al. study.

Finally, a second VA study with 11 years of follow‐up that reviewed cancer deaths among patients with known cancer prior to Regranex exposure (*n* = 477) compared to matched patients without exposure to becaplermin (*n* = 1756) found no difference in the number of cancer deaths (Regranex—87% or 18%, matched comparator—340% or 19%; hazard ratio 0.9, 95% CI 0.7, 1.2), and no suggestion of any dose response. Specifically, in those patients exposed to three or more tubes there was no increased risk (hazard ratio 0.9, 95% CI 0.6, 1.2) [25]. Reports were submitted to FDA by mid 2016, followed by multiple alternative analyses at the Agency's request.

This overall evidence for an absence of increased risk of a new cancer or for a heightened risk of cancer mortality, even with exposure of up to 100–150 daily applications of Regranex onto large open wounds across multiple studies with thousands of patients ultimately led to removal of the Regranex boxed warning in 2018. The extensive time and effort invested in compiling the data and petitioning the FDA for removal of the warning should not be minimised. In fact, the successful removal of the boxed warning from the Regranex label marked the first time a single company accomplished removal of a boxed warning based on clinical evidence and without an advisory committee meeting [31].

## 5 Conclusion

PDGF is an excellent example of the well‐known truism that ‘association’ does not mean ‘causation’. The ‘association’ of elevated levels of endogenous PDGF *and its receptors* in certain types of cancer does not mean that exogenously applied PDGF has the potential to cause cancer; this has never been shown. Constant, constitutive endogenous expression of a ligand and its receptor versus the addition of just the ligand to stimulate tissue regeneration and healing are two fundamentally different cellular processes.

Boxed warnings are placed on products where there is a plausible risk—with an abundance of caution and consistent with the paramount aim of ensuring safety for patients. While boxed warnings found on the labels of hundreds of medical products play an important role in patient safety by highlighting for prescribers (and patients) the potential of an adverse reaction that is serious (e.g., a fatal, life‐threatening or permanently disabling adverse reaction), further rigorous scientific research may prove them to be correct, or erroneous [32]. Removing a boxed warning is an arduous task that requires extensive and robust evidence. The successful removal of the boxed warning from the Regranex label, which only ever pertained to long‐term daily applications onto skin wounds, should be considered from this perspective and should reassure both prescribers and patients of the product's safety even when used daily for up to 20 weeks.

## Conflicts of Interest

S.E.L. is the CEO of Lynch Regenerative Medicine, manufacturer of Regranex. H.B.S. consults for Lynch Regenerative Medicine. The other author declares no conflicts of interest.

## Data Availability

Data sharing not applicable to this article as no datasets were generated or analysed during the current study.

## References

[1] M. Chen , C. Chang , B. Levian , D. T. Woodley , and W. Li , “Why Are There So Few FDA‐Approved Therapeutics for Wound Healing?,” International Journal of Molecular Sciences 24 (2023): 15109, 10.3390/ijms242015109. 37894789 PMC10606455

[2] C. H. Heldin and B. Westermark , “Mechanism of Action and In Vivo Role of Platelet‐Derived Growth Factor,” Physiological Reviews 79 (1999): 1283–1316. 10508235 10.1152/physrev.1999.79.4.1283

[3] H. Li , X. Fu , L. Zhang , Q. Huang , Z. Wu , and T. Sun , “Research of PDGF‐BB Gel on the Wound Healing of Diabetic Rats and Its Pharmacodynamics,” Journal of Surgical Research 145 (2008): 41–48. 18082770 10.1016/j.jss.2007.02.044

[4] S. A. Younan , T. E. Ueland , B. S. Savitz , et al., “Pure Recombinant Platelet‐Derived Growth Factor in Tissue Repair and Rejuvenation: A Review Exploring Frontiers in Regenerative Medicine,” Plast Reconstr Surg, ahead of print, September 15, 2025, 10.1097/PRS.0000000000012426. PMC13007911 40952157

[5] R. Ross , E. W. Raines , and D. F. Bowen‐Pope , “The Biology of Platelet Derived Growth Factor,” Cell 46 (1986): 155–169. 3013421 10.1016/0092-8674(86)90733-6

[6] H. N. Antoniades , T. Galanopoulos , J. Neville‐Golden , C. P. Kiritsy , and S. E. Lynch , “Injury Induces In Vivo Expression of Platelet‐Derived Growth Factor (PDGF) and PDGF Receptor mRNAs in Skin Epithelial Cells and PDGF mRNA in Connective Tissue Fibroblasts,” Proceedings of the National Academy of Sciences of the United States of America 88 (1991): 565–569. 1846446 10.1073/pnas.88.2.565 PMC50852

[7] B. Bucalo , W. H. Eaglstein , and V. Falanga , “Inhibition of Cell Proliferation by Chronic Wound Fluid,” Wound Repair and Regeneration 1 (1993): 181–186. 17163887 10.1046/j.1524-475X.1993.10308.x

[8] B. A. Mast and G. S. Schultz , “Interactions of Cytokines, Growth Factors, and Proteases in Acute and Chronic Wounds,” Wound Repair and Regeneration 4 (1996): 411–420. 17309691 10.1046/j.1524-475X.1996.40404.x

[9] A. B. Wysocki , L. Staiano‐Coico , and F. Grinnell , “Wound Fluid From Chronic Leg Ulcers Contains Elevated Levels of Metalloproteinases MMP‐2 and MMP‐9,” Journal of Investigative Dermatology 101 (1993): 64–68. 8392530 10.1111/1523-1747.ep12359590

[10] N. J. Trengove , M. C. Stacey , S. MacAuley , et al., “Analysis of the Acute and Chronic Wound Environments: The Role of Proteases and Their Inhibitors,” Wound Repair and Regeneration 7 (1999): 442–452. 10633003 10.1046/j.1524-475x.1999.00442.x

[11] G. F. Pierce and T. A. Mustoe , “Pharmacologic Enhancement of Wound Healing,” Annual Review of Medicine 46 (1995): 467–481. 10.1146/annurev.med.46.1.467 7598479

[12] T. A. Mustoe , “Growth Factor‐Induced Acceleration of Tissue Repair Through Direct and Inductive Activities in a Rabbit Dermal Model,” Journal of Clinical Investigation 87 (1991): 694–703. 1991853 10.1172/JCI115048 PMC296361

[13] S. P. Bennett , “Growth Factors in the Treatment of Diabetic Foot Ulcers,” British Journal of Surgery 90 (2003): 133–146. 12555288 10.1002/bjs.4019

[14] M. Cruciani , “Are Granulocyte Colony‐Stimulating Factors Beneficial in Treating Diabetic Foot Infections?: A Meta‐Analysis,” Diabetes Care 28 (2005): 454–460. 15677817 10.2337/diacare.28.2.454

[15] US Food and Drug Administration , “Pre‐Market Approval. GEM 21S Growth Factor Enhanced Matrix,” accessed September 15, 2025, https://www.accessdata.fda.gov/scripts/cdrh/cfdocs/cfpma/pma.cfm?id=P040013.

[16] US Food and Drug Administration , “Pre‐Market Approval. AUGMENT Bone Graft,” accessed September 15, 2025, https://www.accessdata.fda.gov/scripts/cdrh/cfdocs/cfpma/pma.cfm?id=p100006.

[17] T. J. Wieman , J. M. Smiell , and Y. Su , “Efficacy and Safety of a Topical Gel Formulation of Recombinant Human Platelet‐Derived Growth Factor BB (Becaplermin) in Patients With Chronic Neuropathic Diabetic Ulcers. A Phase III Randomized Placebo‐Controlled Double‐Blind Study,” Diabetes Care 21 (1998): 822–827. 9589248 10.2337/diacare.21.5.822

[18] P. A. D'Hemecourt , J. M. Smiell , and M. R. Karim , “Sodium Carboxymethylcellulose Aqueous‐Based Gel vs. Becaplermin Gel in Patients With Nonhealing Lower Extremity Diabetic Ulcers,” Wounds 10 (1998): 69–75.

[19] D. L. Steed , “Clinical Evaluation of Recombinant Human Platelet‐Derived Growth Factor for the Treatment of Lower Extremity Diabetic Ulcers. Diabetic Ulcer Study Group,” Journal of Vascular Surgery 21 (1995): 71–78. 7823364 10.1016/s0741-5214(95)70245-8

[20] J. M. Smiell , T. J. Wieman , D. L. Steed , B. H. Perry , A. R. Sampson , and B. H. Schwab , “Efficacy and Safety of Becaplermin (Recombinant Human Platelet‐Derived Growth Factor‐BB) in Patients With Nonhealing, Lower Extremity Diabetic Ulcers: A Combined Analysis of Four Randomized Studies,” Wound Repair and Regeneration 7 (1999): 335–346. 10564562 10.1046/j.1524-475x.1999.00335.x

[21] T. J. Wieman , “Clinical Efficacy of Becaplermin (rhPDGF‐BB) Gel. Becaplermin Gel Studies Group,” American Journal of Surgery 176, no. 2 (1998): 74S–79S. 10.1016/s0002-9610(98)00185-8 9777976

[22] R. C. Fang and R. D. Galiano , “A Review of Becaplermin Gel in the Treatment of Diabetic Neuropathic Foot Ulcers,” Biologics 2 (2008): 1–12. 19707423 10.2147/btt.s1338 PMC2727777

[23] US Food and Drug Administration , Clinical Review of BLA‐96‐1408 (REGRANEX [Becaplermin] Gel 0.01%) (US Food and Drug Administration, 1997).

[24] L. Ratner , B. Thielan , and T. Collins , “Sequences of the 5′ Portion of the Human *c‐Sis* Gene: Characterization of the Transcriptional Promoter and Regulation of Expression of the Protein Product by 5′ Untranslated mRNA Sequences,” Nucleic Acids Research 15 (1987): 6017–6036. 3627977 10.1093/nar/15.15.6017 PMC306065

[25] U. K. Hull , REGRANEX Gel 0.01% (Becaplermin), Package Insert, Revised (Smith & Nephew, 2018).

[26] N. Ziyadeh , D. Fife , A. M. Walker , G. S. Wilkinson , and J. D. Seeger , “A Matched Cohort Study of the Risk of Cancer in Users of Becaplermin,” Advances in Skin & Wound Care 24 (2011): 31–39. 21173589 10.1097/01.ASW.0000392922.30229.b3

[27] N. Papanas and E. Maltezos , “Benefit‐Risk Assessment of Becaplermin in the Treatment of Diabetic Foot Ulcers,” Drug Safety 33 (2010): 455–461. 20486728 10.2165/11534570-000000000-00000

[28] Y. Rajendran , N. Kondampati , M. Eerike , K. Mali , and L. F. Chalissery , “A Longitudinal Analysis of Black Box Warnings: Trends and Implications for Drug Safety,” Cureus 16 (2024): e57597, 10.7759/cureus.57597. 38706997 PMC11069364

[29] J. S. Yeh , A. Sarpatwari , and A. S. Kesselheim , “Ethical and Practical Considerations in Removing Black Box Warnings From Drug Labels,” Drug Safety 39 (2016): 709–714. 27000800 10.1007/s40264-016-0419-8

[30] D. R. Miller , Becaplermin Use and Cancer Risks in Veterans (American College of Wound Healing and Tissue Repair, 2012).

[31] Smith & Nephew , “Press Release 5 December 2018. FDA Approves Removal of Boxed Warning From REGRANEX (Becaplermin) Gel, 0.01% as Multiple Studies Demonstrated Product Safety,” accessed August 21, 2025, https://www.smith‐nephew.com/en/news/2018/12/05/20181205‐fda‐approves‐removal‐of‐boxed‐warning‐from‐regranex‐gel.

[32] US Food and Drug Administration , Guidance for Industry. Warnings and Precautions, Contraindications, and Boxed Warning Sections of Labeling for Human Prescription Drug and Biological Products—Content and Format (US Food and Drug Administration, 2011).

